# Small Zoom Mismatch Adjustment Method for Dual-Band Fusion Imaging System Based on Edge-Gradient Normalized Mutual Information

**DOI:** 10.3390/s23083922

**Published:** 2023-04-12

**Authors:** Jieling Chen, Zhihao Liu, Weiqi Jin, Jianguo Yang, Li Li

**Affiliations:** MOE Key Laboratory of Optoelectronic Imaging Technology and System, Beijing Institute of Technology, Beijing 100081, China; 3120200518@bit.edu.cn (J.C.); 3120170326@bit.edu.cn (Z.L.); 3120195336@bit.edu.cn (J.Y.); lili@bit.edu.cn (L.L.)

**Keywords:** visible and infrared fusion imaging system, continuous zoom, field-of-view mismatch, edge-gradient normalized mutual information, improved hill-climbing search algorithm

## Abstract

Currently, automatic optical zoom setups are being extensively explored for their applications in search, detection, recognition, and tracking. In visible and infrared fusion imaging systems with continuous zoom, dual-channel multi-sensor field-of-view matching control in the process of synchronous continuous zoom can be achieved by pre-calibration. However, mechanical and transmission errors of the zoom mechanism produce a small mismatch in the field of view after co-zooming, degrading the sharpness of the fusion image. Therefore, a dynamic small-mismatch detection method is necessary. This paper presents the use of edge-gradient normalized mutual information as an evaluation function of multi-sensor field-of-view matching similarity to guide the small zoom of the visible lens after continuous co-zoom and ultimately reduce the field-of-view mismatch. In addition, we demonstrate the use of the improved hill-climbing search algorithm for autozoom to obtain the maximum value of the evaluation function. Consequently, the results validate the correctness and effectiveness of the proposed method under small changes in the field of view. Therefore, this study is expected to contribute to the improvement of visible and infrared fusion imaging systems with continuous zoom, thereby enhancing the overall working of helicopter electro-optical pods, and early warning equipment.

## 1. Introduction

The development of new imaging sensors led to the prospects of their wide applications in multi-band fusion systems [1,2,3,4]. Optical zoom setups are required to satisfy various requirements of an imaging system for search, detection, recognition, and tracking applications. Although the visible-light zoom lens has been extensively used for a long time, most of the early infrared lenses still adopt the prime or dual focus system. Therefore, the early fusion imaging of low-level visible and infrared light is mainly used for night-vision driving assistant systems with a large imaging field of view (FOV), dual-band fusion goggles, and helmets [5]. However, the recent technological breakthrough of the infrared continuous zoom lens has spurred an increasing demand for dual (multiple)-band fusion imaging systems with continuous zoom in unmanned aerial vehicles, helicopter electro-optical pods, photoelectric searches, and early warning equipment [6,7].

**Figure 1:**
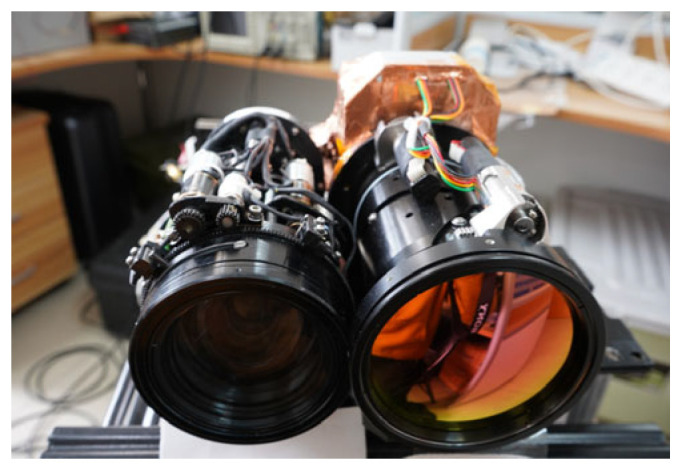
Visible/infrared dual-band fusion imaging system with continuous zoom.

The fusion type in dual-band systems is categorized into multi-sensor fusion. Achieving effective matching depends on a sensitive field-of-view matching similarity measure, an effective autozoom search algorithm, and a fast implementation of the algorithm. For the fixed-focus systems, fast registration and fusion can be achieved by designing a dual-band imaging FOV and pre-calibration [1,8]. For the visible/infrared dual-band fusion imaging systems with continuous zoom (as shown in Figure 1), although the design and pre-calibration can allow the dual-channel field-of-view matching control in the process of synchronous continuous zoom [9], mechanical and transmission errors of the zoom mechanism can still result in a small mismatch of the dual-channel FOV after co-zooming, thus degrading the sharpness of the fusion image and affecting object observation, recognition, and tracking. Therefore, it is necessary to study field-of-view mismatch detection and explore the adjustment methods in dual-band fusion imaging. There are two main solutions for the small mismatch of FOV after continuous co-zooming, either by using image processing methods such as interpolation scaling [10], or by driving the lens to zoom according to the matching relationship of FOV [11]. Since this paper focuses on remote scenes, the use of interpolation scaling will degrade the image quality. Therefore, a conceptual approach to reduce the mismatch in this paper is to drive a particular channel to autozoom according to the field-of-view matching similarity measure between multi-sensor images.

Multi-sensor image matching consists of geometric transformation, similarity measure, and parameter optimization. The selection of similarity measure is an important factor to obtain accurate matching results [12]. The similarity measure can usually be used as the evaluation function of the similarity of multi-sensor field-of-view matching. At present, there are roughly three categories of matching methods for multi-sensor images: region-, feature-, and deep-learning-based [13]. Generally, feature-based methods depend on the selection of feature descriptors, such as points [14,15,16,17,18], edges [19,20,21], regions [22,23], and so on. Wang et al. proposed an improved registration algorithm using double threshold feature extraction and distance disparity matrix [16]. Wang et al. [19] proposed a feature detection and description method based on consistent edge structures of images and then obtained similar contents of infrared images and visible images by detecting consistent edge structures [19]. Heinrich et al. proposed a new method called the modality independent neighborhood descriptor (MIND). The descriptor was used to extract the unique structure of the local neighborhood and calculated it based on the structural differences of adjacent patches [22]. However, it is difficult to generate robust feature descriptors because multi-sensor images are remarkably different, especially in the small mismatch in the FOV. Feature-based methods are also unable to generate a suitable similarity measure function [24]. Moreover, with the development of deep learning, it provides a new idea for multi-sensor image matching. However, deep-learning-based methods require a long execution time and a sufficient number of aligned image pairs for training. It is difficult to do hardware implementation [12,24].

Meanwhile, region-based methods can generate a suitable similarity measure of multi-sensor images. They mainly depend on the gray characteristics of images, which are sensitive to changes in the FOV and have high matching accuracy. These methods are widely used in multi-sensor image registration, especially mutual information (MI) algorithms [25]. Studholme et al. proposed the normalized mutual information (NMI) registration method, which achieved good results in single-mode image registration [26]. However, it can easily fall into the local extremum for multi-sensor image registration. Later, Pluim et al. proposed a gradient normalized mutual information (GNMI) registration method to obtain better results in multi-sensor medical images [27], but it fails to solve the problem related to the weak grayscale correlation between infrared and visible images in natural scenes, especially in the case of small changes in the FOV. Bai et al. proposed a new gradient normalized mutual information (NGNMI) for visible and infrared images, which directly counted the NMI of gradient images [28]. This similarity measure only uses the spatial information of multi-sensor images and cannot obtain the stable single-peak effect. Keshavarz et al. used GNMI as a similarity measure to match the neighborhood of feature points extracted from the edge [29]. It combines point features and GNMI but cannot meet the required operation efficiency. Yang et al. proposed a 4D-CT image sorting method based on mutual information and edge gradient (MIEG), which uses wavelet transform to extract image edges [30]. However, it also fails to solve the problem related to the weak grayscale correlation between infrared and visible images in natural scenes. Krishnan et al. proposed the approach applying a saliency map strategy to convert the infrared and visible face images into the same mode to solve the problem of gray correlation between infrared and visible images [31]. However, it is difficult to find the correct mapping relationship between infrared and visible natural images because of the complexity of natural scenes.

In the autozoom search algorithm, the lens is driven to automatically zoom according to the matching evaluation function of the multi-sensor FOV. The idea is similar to autofocus. Search algorithms mainly include Fibonacci, global search, function approximation, and hill-climbing search algorithms, among others [32,33]. The hill-climbing search algorithm is one of the most commonly used algorithms owing to its simplicity and fast searching. Nevertheless, it is also vulnerable to falling into a local extremum, leading to search failure. Therefore, many studies are devoted to the improvement of this algorithm [34,35,36]. Guo et al. proposed a method selecting the step size and peak according to the slope change in the search process [34]. This method can realize fast search, but the matching evaluation function proposed in this paper has no stable slope change rule. Fu et al. proposed the “three steps” mountain-climb searching algorithm, which adjusted step length according to the position [35]. Jiang et al. proposed a hybrid search method combining hill-climbing search and function approximation algorithms. The small range is determined by the hill-climbing search algorithm, and then the peak is obtained by the function approximation algorithm [36]. These two methods improve the search accuracy to a certain extent, but they are complicated and not conducive to subsequent hardware research.

Although these MI-based similarity measures achieved good results in their corresponding research backgrounds, none of these methods has addressed the problem of weak grayscale correlation between infrared and visible images in natural scenes well, which affects the accuracy of matching similarity evaluation. In addition, infrared and visible images in natural scenes have great similarity in terms of contour edges. Therefore, we enhance the grayscale correlation between infrared and visible images by focusing on the edge images with prominent contour features. Then, the grayscale mutual information of the edge images is combined with the gradient information, and we name this similarity measure as edge-gradient normalized mutual information (EGNMI).

This study entailed the development of a small zoom mismatch adjustment method based on our newly proposed similarity measure, EGNMI, to address the problem of a small mismatch in the FOV in continuous zoom visible/infrared dual-band fusion imaging systems. EGNMI is used as an evaluation function of the field-of-view matching similarity. It solves the problem of poor evaluation results of similar similarity measures (e.g., GNMI) on infrared and visible images with weak grayscale correlation by paying more attention to contour edge features and improving the gradient function. Meanwhile, the traditional hill-climbing search algorithm is improved by using three-frame discrimination and adjusting the search direction change strategy. The improved hill-climbing search algorithm and EGNMI are combined for autozoom, which can suppress the effect of local extremum and search for the best matching point. The comparison experiments of different methods on visible and infrared images of different scenes show that EGNMI is more stable and simplified than other similarity measures, and its combination with the improved hill-climbing search algorithm can effectively accomplish the field-of-view mismatch adjustment.

The remainder of this paper is organized as follows. Section 2 describes the zoom mismatch adjustment method. Section 3 presents the experiments and results analysis. Finally, Section 4 presents the conclusions.

## 2. Materials and Methods

**Figure 2:**
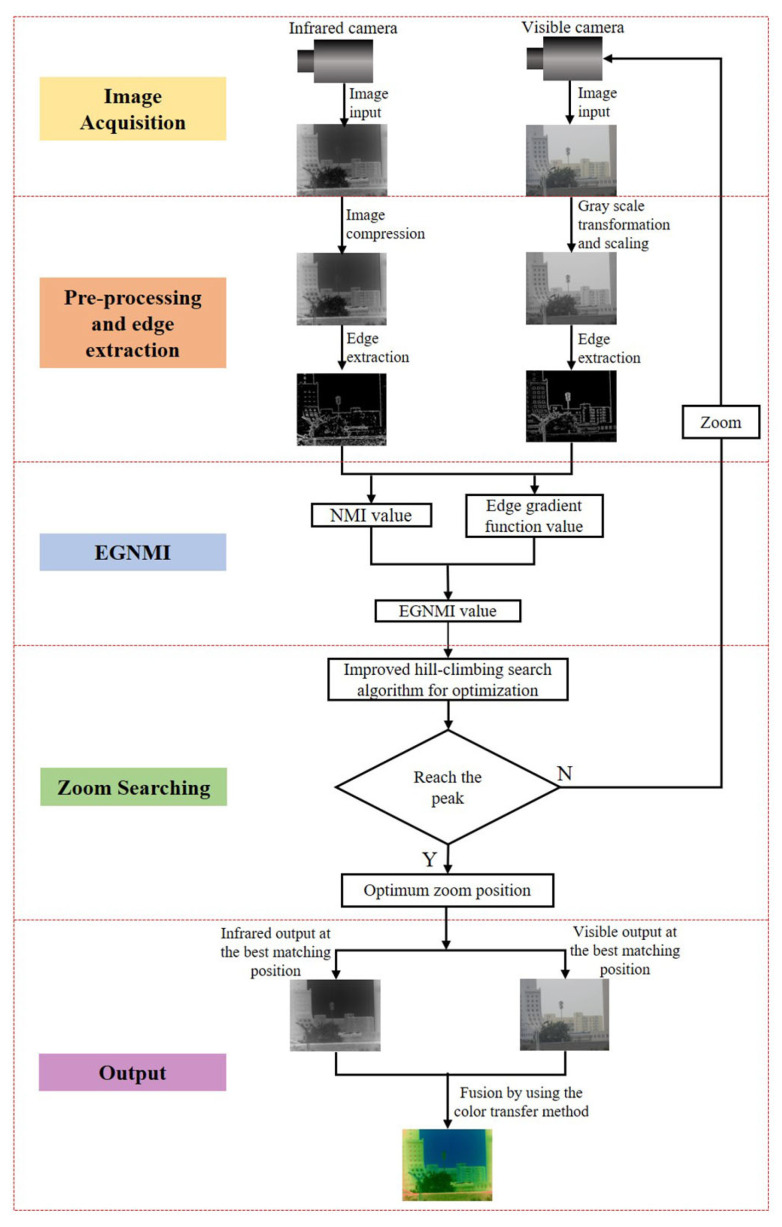
Mismatch adjustment flowchart.

The proposed small zoom mismatch adjustment method for a dual-band fusion imaging system uses EGNMI as the evaluation function of the multi-sensor field-of-view matching similarity. An improved hill-climbing search algorithm is used to adjust the focus of the visible lens to search for the maximum value of the evaluation function (i.e., the best matching position of the multi-sensor FOV). As shown in Figure 2, the initial zoom step size and direction are set for calculating the EGNMI evaluation value between multi-sensor images in the zoom process. The improved hill-climbing search algorithm is used to adjust the zoom step and direction of the visible lens until the maximum value of the evaluation function is determined. Then, the search is stopped, and the best matching of the multi-sensor FOV is achieved. Finally, the infrared and visible images and color fusion images at the best matching position are output.

### 2.1. Evaluation Function Based on Edge Gradient Normalized Mutual Information

#### 2.1.1. GNMI

Multi-sensor images exhibit significant differences in the gray level and main features owing to the varying imaging characteristics of the different sensors used. This makes it difficult to match multi-sensor images based on gray or gradient features separately. It is generally believed that the stability of the similarity evaluation function can be improved by combining gray and gradient information.

For medical images, the GNMI function multiplies gradient information with NMI [27]. It integrates image gray information, magnitude, and direction of the gradient. GNMI is defined as

(1) $$G_{NMI}(A,B)=G(A,B)\times N_{MI}(A,B),$$

where *N_MI_* is the normalized mutual information, and *G* is the gradient function.

*N_MI_* is defined as

(2) $$N_{MI}(A,B)=\frac{H(A)+H(B)}{H(A,B)},$$

where *H*(*A*) and *H*(*B*) are the entropy of the image *A* and *B*, respectively. *H*(*A*,*B*) is the joint entropy of images *A* and *B.*

*G* is defined as

(3) $$G(A,B)=\sum W(\alpha_{A_{ij},B_{ij}}(\sigma ))\times \min(\left|\nabla A_{ij}(\sigma )\right|,\left|\nabla B_{ij}(\sigma )\right|),$$

where *α* is the angle between the gradient vectors, ∇*A_ij_*(*σ*) and ∇*B_ij_*(*σ*) denote the gradient vector of images *A* and *B* at the point (*i*, *j*), respectively, |∙| denotes the magnitude, and *σ* is the standard deviation of the Gaussian function. *W* is the gradient angle function defined by

(4) $$W(\alpha )=\frac{\cos(2\alpha )+1}{2},$$

where the angle *α* can be calculated using the expression below:

(5) $$\alpha_{A_{ij},B_{ij}}(\sigma )=\arccos\frac{\nabla A_{ij}(\sigma )\cdot \nabla B_{ij}(\sigma )}{\left|\nabla A_{ij}(\sigma )\right|\left|\nabla B_{ij}(\sigma )\right|}.$$

To overcome the shortcomings of GNMI, this study developed EGNMI, which investigates the edge images and improves the gradient function.

#### 2.1.2. EGNMI

Unlike GNMI, EGNMI processes infrared and visible edge images. The gradient information of the edge image is used as the weight of the edge NMI to achieve the fusion of gradient and gray information. EGNMI is defined as

(6) $$EG_{NMI}(E_{VIS},E_{IR})=G(E_{VIS},E_{IR})\times EN_{MI}(E_{VIS},E_{IR}),$$

where *E* is the edge image, *EN_MI_*(*E_VIS_*, *E_IR_*) denotes the NMI of visible and infrared edge images, and *G*(*E_VIS_*, *E_IR_*) is the edge gradient function, which considers the magnitude and direction of the gradient.

Compared to MI, NMI has a better overlap invariance. It is normalized by evaluating the ratio of the joint and marginal entropies [26]. Edge normalized mutual information (ENMI) for infrared and visible edge images improves the grayscale correlation between multi-sensor images. *EN_MI_* is defined as

(7) $$EN_{MI}(E_{VIS},E_{IR})=\frac{H(E_{VIS})+H(E_{IR})}{H(E_{VIS},E_{IR})},$$

where *H*(*E_VIS_*) and *H*(*E_IR_*) are the entropy of the visible and infrared edge images, respectively. *H*(*E_VIS_*, *E_IR_*) is the joint entropy of the visible and infrared edge images.

The gradient vector of the image can be calculated by convolving the first derivative of the two-dimensional Gaussian function with the image. Although edge extraction can reduce the feature difference between infrared and visible images to a certain extent, strong gradient information may still exist at a particular position of an image, while the gradient information of another image may be zero or negligible. To reduce the influence of this problem and comprehensively consider the gradient information of both, the gradient function of GNMI (i.e., Equation (3)) was modified in this study. The ratio of the gradient value for each pixel point at the edge of the two images to the total gradient value of the corresponding pixel point of the two images is determined as the coefficient. This coefficient is then multiplied by the gradient values of each other, and the weighted gradient values are further added. When the difference between the two values is large, a small gradient value has a larger proportion. However, for a small difference between the two values, both can be considered. Thus, the gradient function is defined as

(8) $$G(E_{VIS},E_{IR})=\frac{1}{mn}\sum W(\alpha )\frac{2\left|\nabla E_{VIS}{}_{{}_{ij}}\right|\left|\nabla E_{IR}{}_{{}_{ij}}\right|}{\left|\nabla E_{VIS}{}_{{}_{ij}}\right|+\left|\nabla E_{IR}{}_{{}_{ij}}\right|},$$

where *α* is the gradient angle of each pixel in the visible and infrared edge image. *m* and *n* are the length and width of the image, respectively. |∇*E_VISij_*| and |∇*E_IRij_*| are the gradient magnitudes of each pixel in the visible and infrared edge image, respectively, and *W*(·) is the gradient angle function.

To simplify the algorithm complexity, the improved gradient angle function *W* is defined as

(9) $$W(\alpha )=\left|\cos\alpha \right|=\frac{\left|\nabla E_{VIS}{}_{{}_{ij}}\cdot \nabla E_{IR}{}_{{}_{ij}}\right|}{\left|\nabla E_{VIS}{}_{{}_{ij}}\right|\left|\nabla E_{IR}{}_{{}_{ij}}\right|},$$

where ∇*E_VISij_* and ∇*E_IRij_* are the gradient vectors of each pixel in the visible and infrared edge image, respectively.

**Figure 3:**
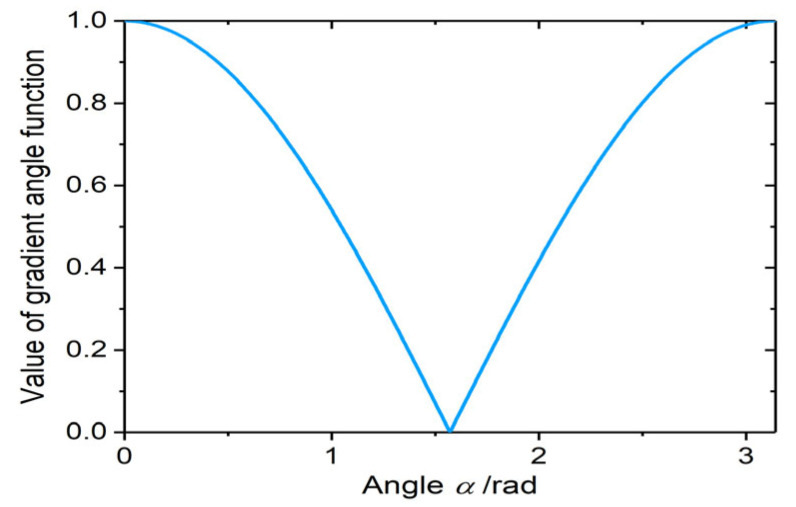
Gradient angle function curve.

Gradients in multi-sensor images for the same pixel position should have either the same or opposite directions. The curve of the improved gradient angle function between 0 and π is shown in Figure 3. If the gradient angle of the corresponding pixels of two images is approximately equal to 0 or π (high coincidence degree of gradient directions), the function value is maximum. If the angle is approximately equal to 90° (low coincidence degree of gradient direction), the function value is minimum.

Substituting Equation (9) into Equation (8), the gradient function *G* can be simplified as follows:

(10) $$G(E_{VIS},E_{IR})=\frac{1}{mn}\sum W(\alpha )\frac{2\left|\nabla E_{VIS}{}_{{}_{ij}}\right|\left|\nabla E_{IR}{}_{{}_{ij}}\right|}{\left|\nabla E_{VIS}{}_{{}_{ij}}\right|+\left|\nabla E_{IR}{}_{{}_{ij}}\right|}=\frac{2}{mn}\sum \frac{\left|\nabla E_{VIS}{}_{{}_{ij}}\cdot \nabla E_{IR}{}_{{}_{ij}}\right|}{\left|\nabla E_{VIS}{}_{{}_{ij}}\right|+\left|\nabla E_{IR}{}_{{}_{ij}}\right|}.$$

### 2.2. Improved Hill-Climbing Search Algorithm

The traditional hill-climbing algorithm first determines the initial search direction and subsequently calculates and compares the evaluation function value of the two images, before and after. If the evaluation function value of the latter image is greater than that of the previous image, the search direction remains the same. Otherwise, the search step size and direction are changed. Although this method has a simple algorithm and strong universality, it is easily affected by local extreme values [32].

**Figure 4:**
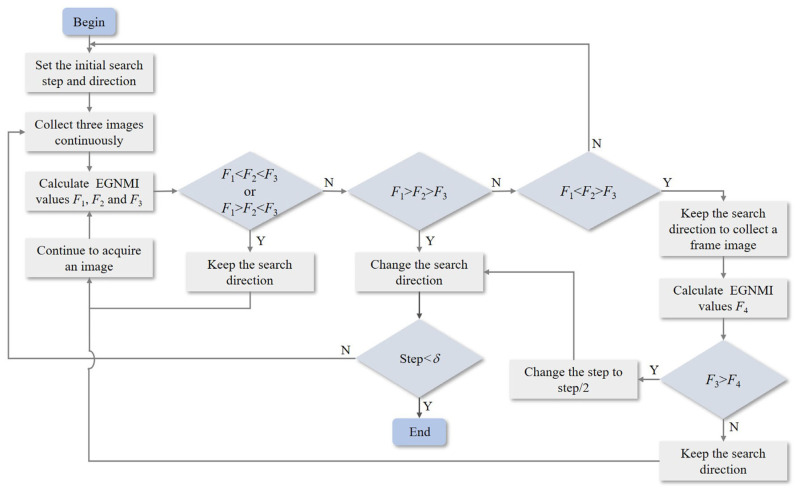
Improved hill-climbing search strategy flowchart.

Although EGNMI has good unimodal properties, some errors still exist, resulting in local maxima (spurious peaks). In the present study, the traditional hill-climbing search algorithm is improved to some extent. The criterion is changed from two to three adjacent images, and the strategy of changing the search direction is improved. When an extreme value is searched, another frame is read and compared to avoid the effect of local extreme values. The flowchart of the improved hill-climbing search algorithm is shown in Figure 4.

The specific steps are as follows:

**Step** **1:** Set the initial search step and direction; then, collect three images continuously. The EGNMI values expressed by *F*_1_, *F*_2_, and *F*_3_ are counted and compared.
**Step** **2:** If *F*_1_ < *F*_2_ < *F*_3_, the search direction is correct; therefore, continue searching. If *F*_1_ > *F*_2_ < *F*_3_, a local minimum point appears. It is recommended to continue searching and observing the direction of the subsequent curve. If *F*_1_ > *F*_2_ > *F*_3_, the search direction is wrong and should be reversed. If *F*_1_ < *F*_2_ > *F*_3_, a peak point exists. To avoid the “spurious peak”, go to Step 3.
**Step** **3:** Maintain the original step and direction to collect a frame image and calculate the EGNMI value expressed as *F*_4_. If *F*_3_ > *F*_4_, change the search direction and change step to step/2; then, continue to search. Otherwise, retain the search direction and avoid this “spurious peak”. When the step is less than the threshold *δ* (the threshold can be determined according to the actual accuracy requirements and system speed), this peak point is considered the best zoom position, corresponding to the maximum point of the evaluation function.

## 3. Experiments and Analysis

**Figure 5:**
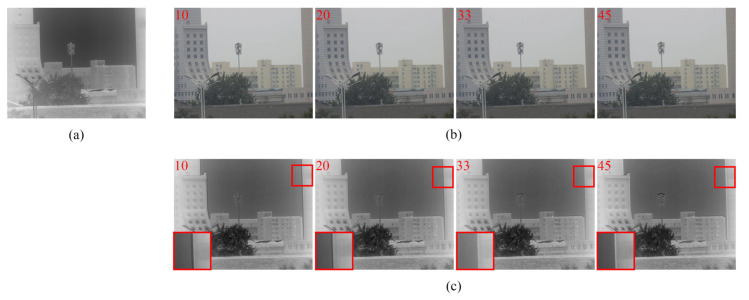
Sequence of pairs of input and fusion images in the zoom process: (**a**) infrared image; (**b**) sequence of visible images; and (**c**) sequence of gray fusion images (the details in red boxes are enlarged to display).

To verify the validity of the method, the image sequence pairs of the fixed-focus infrared lens and the zoom visible lens in different scenes were collected. The dual-band fusion imaging system considered in this study collects 8-bit color visible images with a resolution of 1280 × 1024 pixels and 14-bit infrared images with a resolution of 640 × 512 pixels. We consider a set of 50 image sequence pairs in the zoom process as an example. Figure 5 shows four such pairs (the infrared FOV is almost unchanged because of the fixed-focus infrared lens; therefore, only one is shown here)—frame 10, frame 20, frame 33, and frame 45, where frame 33 is the best matching position. The corresponding infrared and visible images are fused using the color transfer method [37]. In the gray fusion images shown in Figure 5, a slight mismatch at the edges of the buildings can be noticed. The difference is gradually reduced by fine-tuning the focus of the visible lens. After the best matching point, the field-of-view difference gradually increases.

Owing to the differences between the image properties and sizes, the input visible and infrared images were preprocessed. The weighted-average method was used to transform the color visible image into a gray image. The bi-cubic interpolation method was used to scale the visible image into an image with a resolution of 640 × 512 pixels. The infrared image was compressed into an 8-bit image by employing an automatic gain control algorithm.

### 3.1. Edge Extraction

#### 3.1.1. Gradient Constraint-Based Contour Edge Extraction Method

Although there are many differences in detail in the features between visible and infrared images, they share a great deal of similarity in terms of scene contour edges. The extraction of edge images can reduce the impact of natural image complexity and the difference between visible and infrared images. Traditional edge algorithms such as canny [38], LoG [39], and Sobel [40] can extract edge features. The gradient can effectively represent the spatial structure characteristics of the image. The grayscale value change (i.e., gradient value) is larger in the edge part and smaller in the smoother part. The common contour edge features of both infrared and visible images can be extracted by constraining the gradient values. In addition, the EGNMI evaluation function proposed in this paper relies on rich pixel information. Therefore, it is necessary to highlight the contour edge features of infrared and visible images, while retaining more edge pixel information.

This paper proposes a gradient constraint-based edge extraction method. The gradient vector of the image is calculated by convolving the first derivative of the two-dimensional Gaussian function with the image. This study used a 5 × 5 Gaussian gradient mask. Then, Equations (11) and (12) were used to constrain the gradients of the visible and infrared images, respectively, to retain the pixel points larger than the set threshold, thus highlighting the common contour edge features of both.

(11) $$E_{VIS}(x,y)=\left\{\begin{array}{ll} 0 & \left|\nabla I_{VIS}(x,y)\right|<\mu_{VIS}+a\cdot \sigma_{VIS}\\ I_{VIS}(x,y) & \mathrm{other} \end{array}\right.,$$

(12) $$E_{IR}(x,y)=\left\{\begin{array}{ll} 0 & \left|\nabla I_{IR}(x,y)\right|<\mu_{IR}\\ I_{IR}(x,y) & \mathrm{other} \end{array}\right.,$$

where *I_VIS_*(*x*, *y*) and *I_IR_*(*x*, y) are the grayscale values of each pixel of the visible and infrared original images, respectively; *E_VIS_*(*x*, *y*) and *E_IR_*(*x*, *y*) are the grayscale values of each pixel of the visible and infrared edge images, respectively; |∇*I_VIS_*(*x*, *y*)| and |∇*I_IR_*(*x*, *y*)| are the gradient magnitudes of each pixel of the visible and infrared images, respectively; *μ_VIS_* and *μ_IR_* are the average of the visible and infrared image gradient magnitudes, respectively; *σ_VIS_* is its standard deviation; *a* is the coefficient of the standard deviation, which can be assigned according to the desired edge effect.

#### 3.1.2. Comparison of Different Edge Extraction Methods

This section compares the gradient constraint-based contour edge extraction method proposed in this paper with the conventional canny, LoG and Sobel edge detection operators and discusses the contour edge extraction results and the effects of different edge extraction methods on EGNMI.

**Figure 6:**
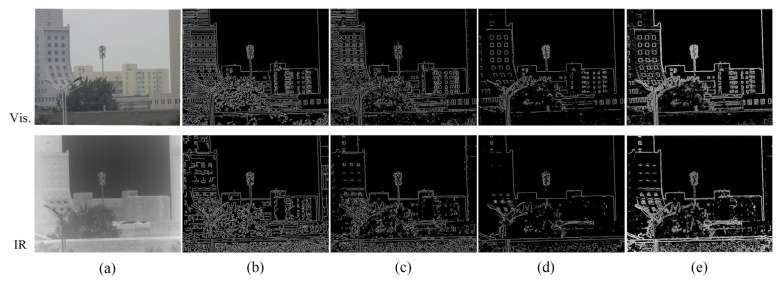
Infrared and visible edge images: (**a**) original; (**b**) canny; (**c**) LoG; (**d**) Sobel; and (**e**) proposed.

**Figure 7:**
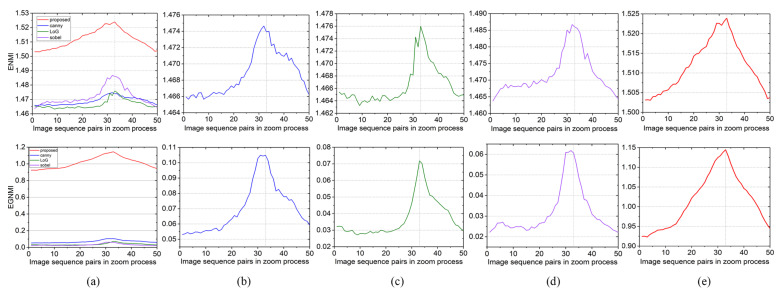
Evaluation function curves based on different edge algorithms: (**a**) overall; (**b**) canny; (**c**) LoG; (**d**) Sobel; and (**e**) proposed.

The edge images obtained by different edge extraction algorithms are shown in Figure 6. The proposed edge extraction approach not only highlights contour edge features well but also retains more pixel information, thus providing a good basis for computing the grayscale MI. Figure 7 shows the ENMI and EGNMI curves of the edge images extracted by different edge algorithms. The ENMI curve demonstrates that the study of edge images can enhance the grayscale correlation between infrared and visible images. Compared with the other three edge extraction methods, the gradient constraint-based contour edge extraction method can better improve the curve quality of EGNMI and obtain a larger range of evaluation function value change.

According to the analysis, the infrared and visible images have large feature similarity at the target scene contour. Although the canny, LoG and Sobel operators all successfully extract detailed edge features, they fail to highlight contour features and retain more pixel information. Unfortunately, many detailed edge features cannot co-exist in the infrared and visible images. Their ENMI values are smaller than those of our method because of less pixel information in the image. When calculating the gradient function values, these features that do not co-exist will be omitted and the co-existing contour features are not highlighted, resulting in small gradient function values. Thus, the EGNMI evaluation values and the range of change are small. The contour edge extraction method we proposed highlights the co-existing contour features and retains more pixel information of the contour edges. These pixels play a large role in calculating the ENMI and gradient function values, which are larger than the other three methods. When the two are multiplied, EGNMI values with a more significant range of change are obtained. Therefore, the gradient constraint-based contour edge extraction method is more suitable for the EGNMI evaluation function.

It is necessary to highlight the contour edge features of infrared and visible images, while retaining more edge pixel information in our research. For further quantitative analysis, we selected the edge entropy EH to analyze the effect of edge algorithms on the degree of retention of image pixel information. We selected the range of function value change *L* and the number of local peaks *β* to analyze the effect of different edge algorithms on the EGNMI evaluation function. EH is the entropy of the edge image, which reflects the degree of pixel information retained by the edge extraction algorithm. A larger value indicates that more pixel information is retained. It is defined as

(13) $$EH(E)=-\sum_{l}P_{E}(l)\log P_{E}(l),$$

where *E* is the edge image. *l* is the grayscale value of the edge image. *P_E_*(*l*) is the probability of grayscale distribution.

*β* is the number of local peaks of the evaluation function curve. The smaller the value of *β*, the better the unimodal property of the evaluation function and the stronger the anti-interference ability. The range of function value change *L* shows the influence of the pixel information retained by the edge extraction algorithm on the EGNMI. It is defined as

(14) $$L=f_{\max}-f_{\min},$$

where *f_max_* and *f_min_* correspond to the maximum and minimum values of the evaluation function, respectively.

Table 1 shows the comparison of the edge entropy EH, range of function value change *L,* and number of local peaks *β* of the four edge algorithms. *L* and *β* are not counted for wrong matches. Compared to canny, LoG, and Sobel, the proposed highlights the contour edge features and retains more pixel information, resulting in a larger range of function value changes. The EGNMI evaluation function based on the proposed edge extraction method exhibits better unimodality. Therefore, the gradient constraint-based contour edge extraction method is more suitable for the EGNMI evaluation function.

#### 3.1.3. Parameter Setting and Analysis

Visible images tend to have richer detail features than infrared images. Therefore, an additional standard variance component is added to the contour edge extraction of visible images, thus highlighting the co-existing contour edge features of infrared and visible images. The coefficient of the standard deviation *a* can affect the contour edge extraction effect of visible image. This section will discuss the selection of the coefficient *a* and analyze the effect on the EGNMI for different values of *a*.

**Figure 8:**
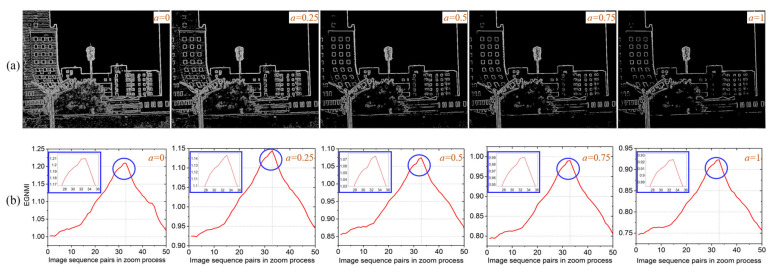
Experimental results under different values of *a*: (**a**) edge images under different values of *a*; and (**b**) EGNMI curves under different values of *a* (the details in blue circles are enlarged to display).

The cases where *a* is 0, 0.25, 0.5, 0.75, and 1 are selected for analysis and comparison, respectively. Figure 8 shows the edge extraction results and EGNMI curves for these five values. From Figure 8a, it can be found that detailed edge features and the number of pixel points near contour edges gradually decreases as the value of *a* gradually increases. Figure 8b shows EGNMI curves under different values of *a*. On the whole, good EGNMI evaluation results have been obtained. When *a* = 0.25, the best quality of the EGNMI curve has been achieved, which has the most obvious peak at the best matching position.

To further quantitatively compare these five cases, we still select edge entropy EH, range of function value change *L,* and number of local peaks *β* for analysis. Table 2 shows the results of EH, *L*, and *β* under different values of *a*. When *a* = 0, the edge image contains the most pixel information. When *a* = 0.25, the best quality of the EGNMI curve is obtained.

According to the analysis, when *a* = 0, although it has the most pixel information and prominent contour edges, it contains too many detailed edges that infrared edge images do not have, which affects the quality of the EGNMI curve. While the edge images with *a* of 0.5, 0.75, and 1 still highlight the main contour edge features, the number of pixel points near the contours is significantly reduced. The reduction in the effective pixel points involved in the EGNMI calculation affects the results of the evaluation function. When *a* = 0.25, it maintains the balance of highlighting common contour edges and including more pixel information, resulting in the best evaluation results of EGNMI. To conclude, the gradient constraint-based contour edge extraction method combined with EGNMI can obtain good evaluation results, especially when *a* = 0.25, the best evaluation results can be obtained.

### 3.2. Evaluation and Search Results for Different Scenes

**Figure 9:**
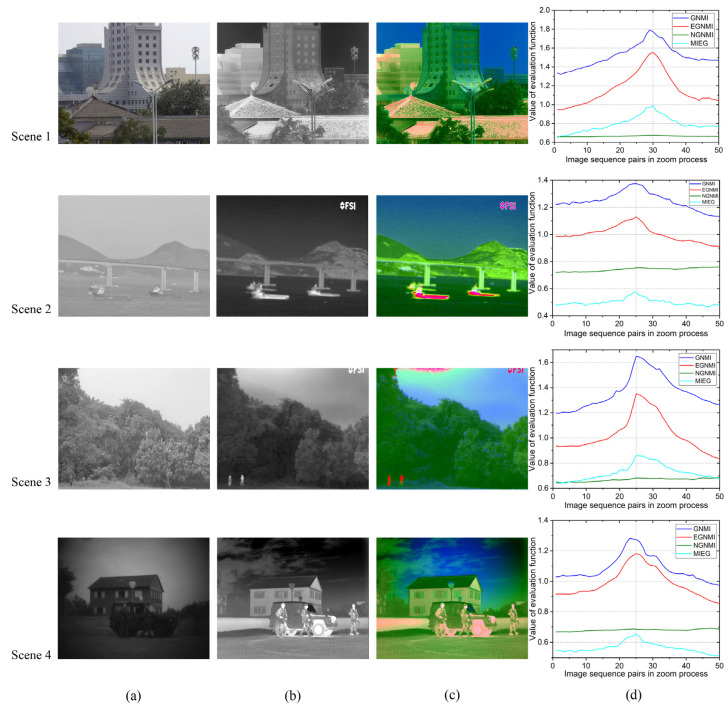
Results of different scenes based on different evaluation functions: (**a**) visible images; (**b**) infrared images; (**c**) color fusion images; and (**d**) evaluation function comparison.

To further verify the feasibility and universality of this algorithm, we constructed a dataset of images with small mismatches in the two-channel FOV. For remote scene imaging with a fusion imaging system, the translation parallax can be ignored in the dual-channel parallel optical path. The matching of different fields of view in the zoom process is mainly affected by magnification. Therefore, the dataset mainly contains image sequence pairs of real zoomed visible and fixed-focus long-wave infrared images, as well as the image sequence pairs obtained by simulating the zoom at 0.0005 magnification steps based on the originally registered image pairs. In this study, four types of actual scenes were selected (as shown in Figure 9). Scene 1 mainly consists of buildings, a blue sky, and trees. Scene 2 consists of a blue sky, mountains, water, bridges, and boats. Scene 3 mainly consists of a blue sky, trees, and people. Finally, Scene 4 consists of a blue sky, buildings, cars, and people. The image pairs of Scene 1 are obtained by the real zoom visible lens and fixed-focus infrared lens. The fusion experiment shows that the 30th image pair is the best match. The 50 sequence image pairs of Scenes 2, 3, and 4 are obtained by simulating the zoom based on the originally registered image pairs (these images are from the TNO Image Fusion Dataset and VLIRVDIF). The 25th pair of images is set as the best match.

Figure 9 shows the visible, infrared, and fusion images at the best matching point and the comparison of evaluation function curves of EGNMI, GNMI, NGNMI, and MIEG in the zoom process. The horizontal coordinate of Figure 9d denotes the infrared and visible image pairs during the zoom process, and the vertical coordinate denotes the evaluation values of the degree of field-of-view matching obtained by different evaluation functions.

**Figure 10:**
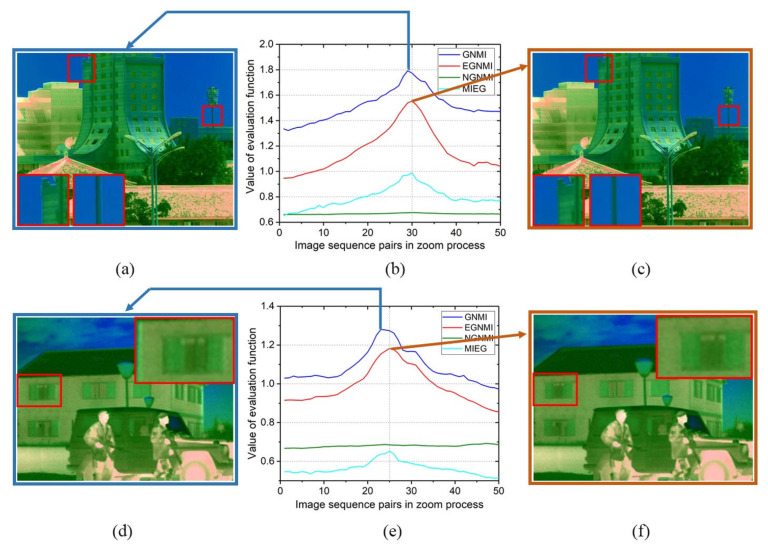
Fusion image matching comparison of different peak values in two scenes (the details in red boxes are enlarged to display): (**a**) frame 29 in Scene 1; (**b**) evaluation function in Scene 1; (**c**) frame 30 in Scene 1; (**d**) frame 23 in Scene 4; (**e**) evaluation function in Scene 4; and (**f**) frame 25 in Scene 4.

In Scene 1, EGNMI, MIEG, and NGNMI determine frame 30 as the best matching point, while GNMI incorrectly determines frame 29. Figure 10a–c verify this result. Evidently, as the edges of the building match better in frame 30 than in frame 29, the two fields of view match better in frame 30 than in frame 29. The value of NGNMI has little change compared with other evaluation functions. In Scene 2, GNMI, EGNMI, and MIEG identify frame 25 as the best matching point, while NGNMI makes mistakes. It has no proper curve trends, and the best matching point is not achieved at the maximum value. In Scene 3, GNMI, EGNMI, and MIEG identify frame 25 as the best matching point, while NGNMI makes mistakes. In Scene 4, EGNMI and MIEG identify frame 25 as the best matching point, while GNMI and NGNMI make mistakes. GNMI incorrectly determines frame 23, and NGNMI has no proper curve trends. Figure 10d–f show that frame 25 has a significantly better field-of-view match than frame 23. Therefore, the EGNMI method can consistently obtain the best frame. Compared with GNMI, NGNMI, and MIEG, which have many local extreme points, EGNMI demonstrates better unimodality and stability. Moreover, EGNMI performs better in detail-rich scenes than in large open scenes, such as lakes and skies.

**Figure 11:**
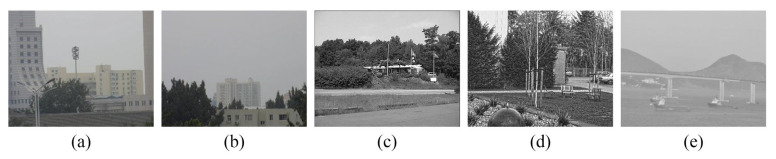
Five sets of scenes chosen for demonstrating the effectiveness of the evaluation function and search algorithm: (**a**) urban scene; (**b**) urban scene with a large open view; (**c**) forest scene; (**d**) park scene; (**e**) bay scene.

To demonstrate the effectiveness of the evaluation function and its search algorithm, five sets of scenes were selected (as shown in Figure 11). Table 3 shows the search points and the correctness statistics of the search results based on the traditional and improved hill-climbing search algorithms for NMI, GNMI, NGNMI, MIEG, and EGNMI (Y for matching and N for not matching). The following inferences can be drawn from the results shown in Table 3. (i) Evaluation functions based on the traditional hill-climbing search algorithm can easily fall into local extremum, even at the initial stage. By contrast, EGNMI has better search accuracy, but it may still fall into local extremum. (ii) Evaluation functions based on the improved hill-climbing search algorithm can suppress the effect of local extremes, and the search accuracy remains unchanged or is improved to some extent. EGNMI can accurately find the best matching position.

The main reason for the incorrect searching is that the best matching point of the evaluation function is incorrect, or too many local extreme points around the best matching point affect the discrimination of the maximum value. Therefore, EGNMI based on the improved hill-climbing search algorithm has the best accuracy and stability in the comprehensive evaluation.

We selected the sensitivity *M*, the number of local peaks *β*, and the algorithm time *τ* to quantitatively analyze the performance of GNMI, NGNMI, EGNMI, and MIEG based on the improved hill-climbing search algorithm. The algorithm time *τ*, which reflects the processing speed of the algorithm, is the time required to evaluate an image frame. The sensitivity *M* represents the intensity of changes of the field-of-view matching evaluation function near the maximum value [41]. It reflects the sensitivity of the evaluation function in the small field-of-view changes. The larger the value, the higher its sensitivity. It is defined as

(15) $$M=\frac{f_{max}-f(x_{max}+\Delta x)}{f(x_{max}+\Delta x)},$$

where *f_max_* is the maximum value, and *f*(*x_max_* + Δ*x*) is the value of the function after the abscissa *x_max_* at the maximum value of the evaluation function changes by Δ*x*. In this study, Δ*x* would have the value of 4 or −4, depending on the coordinates where the function value is larger.

Table 4 presents the comparison of *M*, *β*, and *τ* of the four field-of-view evaluation functions. They are simulated on the MATLAB platform under five groups of scenes. *M* and *β* are not counted for incorrect matches. EGNMI has better field-of-view sensitivity and unimodal properties. The aforementioned qualitative analysis of function curves is further verified. Although the real-time performance of EGNMI is slightly unsatisfactory compared to that of NGNMI, compared to GNMI and MIEG, EGNMI improves the processing speed of the algorithm by operating on edge images and simplifying the gradient function. NGNMI has the best real-time performance compared to the other three algorithms because it only considers gradient information. However, its poor unimodal property and sensitivity can easily lead to search errors. The EGNMI method takes approximately 15 frames and 1.5 s to search for the best matching position. If a hardware system such as FPGA or GPU is used for parallel processing, and the processing algorithm flow is further optimized, the small mismatch adjustment can be completed within the processing time of 3–5 frames. Therefore, it can meet the demand of practical applications.

### 3.3. Noise Robustness Experiment

**Figure 12:**
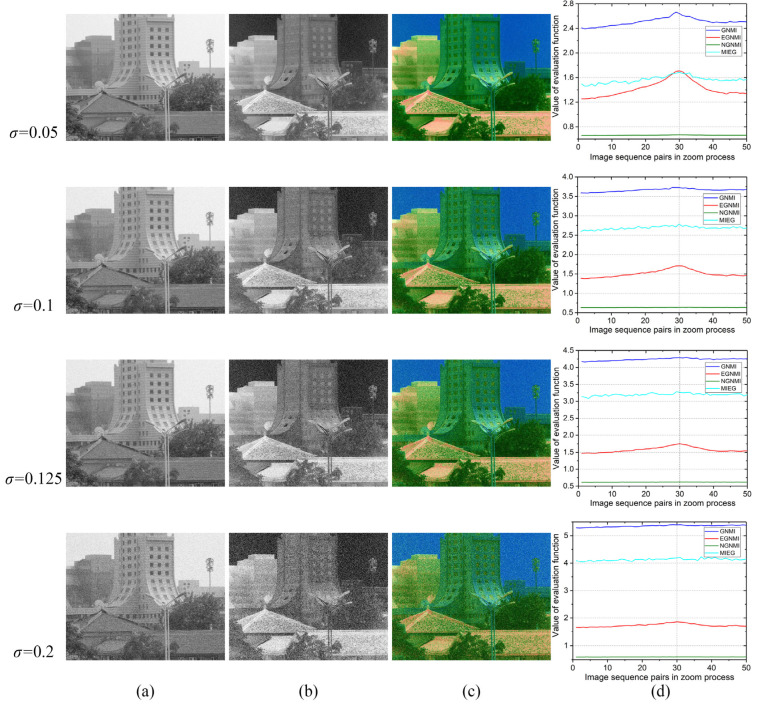
Results of noise experiments: (**a**) visible images; (**b**) infrared images; (**c**) color fusion images; and (**d**) evaluation function comparison.

Noise experiments were conducted to verify the robustness of EGNMI against noise. Gaussian noise with a standard deviation *σ* of 0.05, 0.075, 0.1, 0.125, 0.15, 0.175, and 0.2 was added to the visible and infrared sequential images and repeated 10 times. There were 70 sets of noisy images in total. We compared the curves of different evaluation functions under noise with different standard deviation. Meanwhile, the noise robustness of the evaluation function was verified by observing the quality of the function curve under repeated superposition of noise. Figure 12 shows the results of a set of experiments under noise with different standard deviation. The curves of GNMI, EGNMI, NGNMI, and MIEG under noise with a standard deviation of 0.05, 0.1, 0.125, and 0.2 are shown. The 30th image pair is identified as the best match.

The results of this group of experiments indicate that the quality of the evaluation function curves decreases to some extent with the addition of Gaussian noise. When *σ* = 0.125, GNMI, NGNMI, and MIEG deviate from the original maximum point, whereas EGNMI maintains its original maximum point and has a better curve quality. When *σ* = 0.2, EGNMI still maintains the original maximum point.

**Figure 13:**
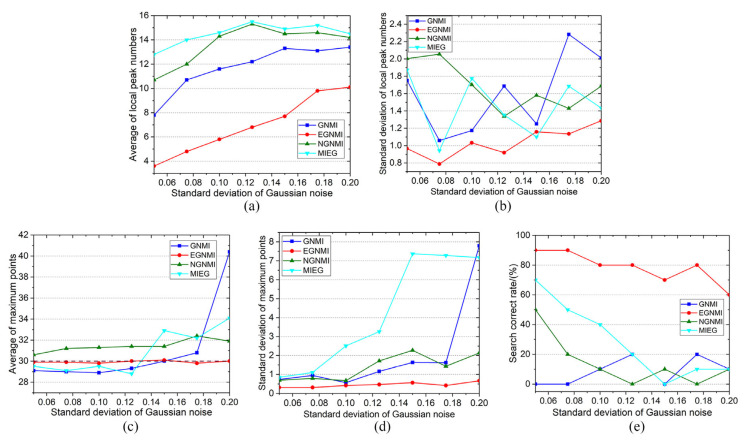
Noise robustness evaluation: (**a**) average of local peak numbers; (**b**) standard deviation of local peak numbers; (**c**) average of maximum points; (**d**) standard deviation of maximum points; and (**e**) search correct rate.

Under this set of experimental results (shown in Figure 12), although the quality of other evaluation functions decreases under the influence of noise, EGNMI can maintain a better curve quality and its original maximum point. We used the average and standard deviation of the number of local peaks, the average and standard deviation of the maximum points, and the search correct rate to quantitatively verify the noise robustness of the evaluation functions. Figure 13 shows the comparison of the maximum point, number of local peaks, and search correct rate of evaluation function curves under noise with a standard deviation of 0.05, 0.075, 0.1, 0.125, 0.15, 0.175, and 0.2. As shown in Figure 13a,b, the curve quality of EGNMI is more stable, and EGNMI exhibits the best unimodal property in the repeated noise superposition experiments. Figure 13c,d show that EGNMI has the best stability of maximum points, and its average maximum point is closest to the best match. Figure 13e shows that EGNMI has the highest search success rate. Thus, it is quantitatively verified that EGNMI can better adapt to the effects of noise. EGNMI can be used to study gradient and grayscale correlation on edge images to reduce the effect of noise. Furthermore, the edge extraction algorithm in this study is robust to noise. Therefore, EGNMI has the best noise robustness compared to GNMI, NGNMI, and MIEG.

In summary, compared to NMI, GNMI, NGNMI, and MIEG, EGNMI has the best unimodal property, scene universality, accuracy, sensitivity, and noise robustness for the case of a small mismatch in the infrared and visible FOV. The real-time performance of EGNMI is better than GNMI and MIEG, thus making it suitable for practical applications.

## 4. Conclusions

In this study, we investigated the combination of the multi-sensor image registration and automatic zoom for the correction of a small mismatch in the FOV, which is caused by mechanical and transmission errors of the zoom mechanism in the zoom process of the visible/infrared dual-band fusion imaging systems. After continuous co-zooming, the visible lens is driven to autozoom according to the matching evaluation function. Thus, this study devised an adjustment method for small zoom mismatch for a dual-band fusion imaging system based on EGNMI. EGNMI combines edge mutual and edge gradient information. The proposed method uses it as the evaluation function of the matching similarity of the multi-sensor FOV. Furthermore, the gradient function, which is sensitive to small changes in the FOV, is constructed to reduce the complexity of the algorithm. Then, the improved hill-climbing search algorithm and EGNMI evaluation function are combined for autozoom. The experimental findings demonstrate that EGNMI has the best unimodal property, scene universality, accuracy, sensitivity, and noise robustness compared to other methods (i.e., NMI, GNMI, NGNMI, and MIEG). The real-time performance of EGNMI is better than GNMI and MIEG and is thus suitable for practical applications. The improved hill-climbing search algorithm can effectively suppress the effect of local extremum and search for the best matching point.

The proposed method solves the problem of the small mismatch in the FOV of the continuous zoom fusion system. The hardware processing research of the algorithm is currently in progress and is expected to extend the application of continuous zoom fusion systems into new areas, including unmanned aerial vehicles (UAVs)/helicopter electro-optical pods, photoelectric search, early warning equipment, security monitoring, and various other fields. The system can search for the target in a large range and track the target in a small range, simultaneously ensuring the clarity of the fusion image.

## Figures and Tables

**Table 1:** Comparison of different edge algorithms.

Edge Algorithm	EH(VIS)	EH(IR)	*L*	*β*
Canny	0.9564	1.0717	0.0514	8
LoG	0.7993	0.6834	0.0447	5
Sobel	0.3399	0.3758	-	-
Proposed	**1.8842**	**1.7943**	**0.2220**	**0**

**Table 2:** Comparison of edge extraction under different values of *a*.

*a*	EH	*L*	*β*
0.00	**2.6949**	0.1980	2
0.25	1.8842	**0.2220**	**0**
0.50	1.3381	0.2191	3
0.75	1.1782	0.1964	1
1.00	0.9784	0.1769	2

**Table 3:** Comparison of evaluation functions based on traditional and improved hill-climbing search algorithms.

Image Group Number and Best Matching Point		Searching Method	NMI		GNMI		NGNMI		MIEG		EGNMI	
			Match or Not	Search Point	Match or Not	Search Point	Match or Not	Search Point	Match or Not	Search Point	Match or Not	Search Point
a	33	traditional	N	8	N	1	N	5	N	3	Y	33
		improved	N	29	Y	33	N	37	Y	33	Y	33
b	24	traditional	N	3	N	5	Y	24	N	26	Y	24
		improved	N	33	N	26	Y	24	N	26	Y	24
c	25	traditional	N	1	N	24	N	1	N	3	Y	25
		improved	N	23	N	23	Y	25	N	24	Y	25
d	25	traditional	N	5	Y	25	Y	25	N	1	Y	25
		improved	N	31	Y	25	Y	25	Y	25	Y	25
e	25	traditional	N	1	N	3	N	3	N	5	N	2
		improved	N	30	Y	25	N	37	Y	25	Y	25
Search correct rate		traditional	0		20%		40%		0		80%	
		improved	0		60%		60%		60%		100%	

Y for matching and N for not matching.

**Table 4:** Comparison of evaluation functions at different scenes.

Image Group Number	*M*				*β*				*τ*/ms			
	GNMI	NGNMI	MIEG	EGNMI	GNMI	NGNMI	MIEG	EGNMI	GNMI	NGNMI	MIEG	EGNMI
a	0.0170	-	0.0207	**0.0237**	13	-	12	**0**	145.96	**71.23**	908.33	91.47
b	-	0.0010	-	**0.0170**	-	7	-	**0**	146.56	**64.34**	941.91	92.15
c	-	0.0028	-	**0.0328**	-	12	-	**1**	113.28	**67.45**	841.26	82.48
d	0.0495	0.0081	0.0421	**0.0533**	6	9	11	**1**	128.11	**72.26**	837.51	90.12
e	0.0306	-	0.0666	**0.0715**	6	-	13	**3**	116.39	**66.49**	885.08	84.14

## Data Availability

Not applicable.
